# In vivo assessment of the drug interaction between sorafenib and paracetamol in rats

**DOI:** 10.1007/s00280-020-04075-3

**Published:** 2020-05-11

**Authors:** Agnieszka Karbownik, Katarzyna Sobańska, Tomasz Grabowski, Joanna Stanisławiak-Rudowicz, Anna Wolc, Edmund Grześkowiak, Edyta Szałek

**Affiliations:** 1 Department of Clinical Pharmacy and Biopharmacy, Poznań University of Medical Sciences, 14 Św. Marii Magdaleny Str., 61-861 Poznań, Poland; 2 Polpharma Biologics SA, Trzy Lipy 3 Str., 80-172 Gdańsk, Poland; 3 Univeristy Hospital of Lord’s Transfiguration, ul. Szamarzewskiego 84/86, Poznań, Poland; 4 Department of Animal Science, Iowa State University, 239E Kildee Hall, Ames, IA 50011 USA; 5 Hy-Line International, 2583 240th Street, Dallas Center, IA 50063 USA

**Keywords:** Sorafenib, Sorafenib *N*-oxide, Paracetamol, Paracetamol glucuronide and paracetamol sulphate, Pharmacokinetics, Drug–drug interaction

## Abstract

**Purpose:**

Sorafenib is a multi-targeted tyrosine kinase inhibitor (TKI) used for the treatment of advanced renal cell carcinoma, hepatocellular carcinoma and radioactive iodine resistant thyroid carcinoma. Neoplastic diseases are the cause of pain, which may occur regardless of the stage of the disease. Paracetamol is a non-opioid analgesic used alone or in combination with opioids for the treatment of cancer pain. Numerous studies have pointed out changes in the pharmacokinetic parameters of TKIs when co-administered with paracetamol. The aim of the study was to assess drug–drug interactions (DDIs) between sorafenib and paracetamol.

**Methods:**

Rats were divided into three groups, each consisting of eight animals. The first group received sorafenib (II_S_), the second group received sorafenib + paracetamol (I_S+PA_), whereas the third group received only paracetamol (III_PA_). A single dose of sorafenib (100 mg/kg b.w.) and paracetamol (100 mg/kg b.w.) was administered orally. The plasma concentrations of sorafenib and its metabolite–N-oxide as well as paracetamol and its glucuronide and sulphate metabolites were measured using validated high-performance liquid chromatography (HPLC) method with ultraviolet detection.

**Results:**

The co-administration of sorafenib and paracetamol increased the maximum concentration (*C*_max_) of paracetamol by 33% (*p* = 0.0372). In the *I*_S+ PA_ group the *C*_max_ of paracetamol glucuronide was reduced by 48% (*p* =  < 0.0001), whereas the *C*_max_ of paracetamol sulphate was higher by 153% (*p* = 0.0012) than in the III_PA_ group. Paracetamol increased sorafenib and sorafenib *N*-oxide *C*_max_ by 60% (*p* = 0.0068) and 83% (*p* = 0.0023), respectively.

**Conclusions:**

A greater knowledge of DDI between sorafenib and paracetamol may help adjust dose properly and avoid toxicity effects in individual patients.

## Introduction

According to epidemiological data, morbidity and mortality rates due to malignant tumors are continuously increasing. Chemotherapy may be an effective systemic treatment of cancer, but this method has many limitations resulting especially from tumour heterogeneity and acquired cancer cells resistance [1]. Moreover, due to insufficient selectivity of drugs and their multidirectional mechanism of action, there is still a problem of serious and life-threatening adverse drug reactions [2]. They contribute to reduction of the quality of patients’ life and ultimately may lead to the failure of the therapy. Another issue of anticancer therapy is potential for drug–drug interactions (DDIs). Today’s chemotherapy is frequently based on concomitant administration of classic chemotherapeutics, biologic agents, targeted drugs as well as many types of adjuvant therapies. These complex combinations may increase pharmacokinetic and pharmacodynamic interactions that can negatively influence the treatment safety and efficacy [2, 3]. Therefore, it is important to investigate drug combinations that may be relevant in clinical practice.

Tyrosine kinase inhibitors (TKIs) are a dynamically growing group of small-molecule agents that play a crucial role in today’s oncology. Sorafenib (Nexavar^®^) is one of the promising members of TKIs. It has been used to treat adult patients with advanced renal cell carcinoma (RCC) after failure of prior treatment with tyrosine kinase inhibitors (sunitinib, pazopanib–first-line treatment), since 2005 [4, 5]. It has also been used to treat patients with hepatocellular carcinoma (HCC), since 2007 [6], and finally in patients with progressive, locally advanced or metastatic, differentiated (papillary/follicular/Hürthle cell) thyroid cancer, resistant to treatment with radioactive iodine, since 2013 [7]. Additionally, the efficacy of the drug has been assessed in many clinical trials conducted on adults with various types of cancer, including breast cancer, salivary gland cancer, melanoma, non-small cell lung cancer, glioma [8–13]. Nowadays the drug is mostly used to treat hepatocellular carcinoma (HCC) due to limited possibilities of treatment of the advanced stages of this cancer [14, 15].

The mechanism of action of sorafenib includes blocking cellular signal transduction by binding to the intracellular domains of membrane receptors, which belong to the tyrosine kinase group, i.e.: VEGFR-1, VEGFR-2, VEGFR-3, PDGFR-β, FLT-3, Kit, FGFR1 and RET. Sorafenib also inhibits serine/threonine kinases, such as B-Raf and Raf1 [16–18]. Like regorafenib, sorafenib is a kinase inhibitor not only metabolized by CYP450, but also transformed by UDP-glucuronyl transferases [19]. Sorafenib is a substrate of phase I and II enzymes and may interact with other drugs that undergo these metabolic pathways. Sorafenib also inhibits some UGT isoforms [20]. In vitro studies showed that it particularly inhibits UGT1A1 and UGT1A9 [21].

Pain appears in 55% of patients undergoing anticancer therapy and in 66% of patients at the advanced, metastatic and terminal stages of cancer [22]. Paracetamol (acetaminophen) is the most common painkiller in Step I of the WHO analgesic ladder [23]. It is mostly metabolized in the liver, where it is conjugated with glucuronic (60%), sulphuric (30%) acids and with cysteine (3%). The reaction is catalysed by glucuronyl transferase and sulfotransferase. A small amount, i.e. about 5–10%, is *N*-hydroxylated through cytochrome P-450 (CYP2E1) to toxic *N*-acetyl-*p*-benzoquinone imine (NAPQI) [24]. NAPQI is highly reactive compound that is mainly responsible for paracetamol-induced hepatotoxicity. NAPQI is further detoxified in a nonenzymatic reaction with sulfhydryl groups of glutathione and the resulting compound is ultimately excreted with the urine as cysteine and mercapturic acid conjugates [25].

As it is necessary to apply painkillers in oncological therapy, the likelihood of simultaneous administration of sorafenib and paracetamol increases. Taking into account the fact that sorafenib strongly inhibits some UGTs [26] and that glucuronidation is one of the metabolic pathways of paracetamol, there is a risk of interaction between these two drugs. Therefore, the aim of this study was to assess the potential for interaction between sorafenib and paracetamol.

## Materials and methods

### Reagents

Sorafenib (CAS number 284461-73-0), sorafenib *N*-oxide, paracetamol glucuronide and paracetamol sulphate were purchased from LGC Standards (Łomianki, Poland). Lapatinib (CAS number 231277-92-9), paracetamol (CAS number 103-90-2), methanol, acetonitrile, sodium sulphate, ethyl acetate, glacial acetic acid, formic acid, perchloric acid, theophylline, ammonium acetate, sodium hydroxide and dimethyl sulfoxide (DMSO) were purchased from Sigma-Aldrich (Poznań, Poland). Water used in the mobile phase was deionised, distilled and filtered through a Millipore system (Direct Q3, Millipore, USA) before use. Sorafenib tosylate (Nexavar^®^, batch number BXHT61) was purchased from Bayer Polska Sp. z o.o. (Warsaw, Poland). Paracetamol (Pedicetamol, batch number K003) was purchased from Sequoia sp. z o.o. (Warsaw, Poland).

### Animals

The experimental protocol assuming involvement of animals in this study was reviewed and approved by the Local Ethics Committee, Poznań, Poland (no 61/2017). All procedures were performed in accordance with the European Union regulations concerning the handling and use of laboratory animals. Adult male Wistar rats (weight 480–530 g) were used in the study. The animals were maintained under standard breeding conditions with a 12/12 h light–dark cycle (lights on at 06.00, lights off at 18.00) at constant room temperature (23 ± 2 °C), relative humidity of 55 ± 10% and given ad libitum access to food and water. The animals were allowed to acclimatise for a week before the beginning of the experiments. The rats were divided into three groups. One group received sorafenib and paracetamol (*I*_S+PA_), another group received sorafenib (II_S_), whereas the last group received paracetamol (III_PA_). Sorafenib (100 mg/kg b.w [27]) was dissolved in 1 mL 10% DMSO and administered directly into the animals’ stomachs using a gastric probe. To make sure that the animals received the entire dose of the drug, 1 mL of 10% DMSO was then administered to rinse the probe. 100 µL of blood was collected from each rat by cutting off a piece of his tail. The blood samples were collected into heparinised test tubes at the following time points: 0, 0.5, 1, 2, 3, 4, 5, 6, 7, 8, 10, 12, 24, 30, 48, 72, 96 h. Paracetamol was administrated as an oral solution (Pedicetamol^®^, 100 mg/ml) at a dose of 100 mg/kg b.w [28]. to the *I*_S+PA_ and III_PA_ groups. Blood samples for paracetamol analysis were collected before (0′) and 5, 15, 30, 60, 90, 120, 240, 360 and 480 min after the drug administration. All the blood samples were centrifuged at 2.880 g for 10 min at 4 °C.

### HPLC–UV assay

The concentrations of paracetamol, paracetamol glucuronide and paracetamol sulphate were assayed using the high-performance liquid chromatography (HPLC) method with ultraviolet (UV) detection [29]. Separation was achieved by isocratic elution of the mobile phase, comprising sodium sulphate 0.05 M pH 2.2 (adjusted with 85% orthophosphoric acid) and acetonitrile (93:7, v/v), at a flow rate of 1.0 mL/min through an BDS Hypersil^®^ C18 column (150 mm × 4.6 mm, 5.0 μm particle size) (Thermo Electron Corporation^®^, Waltham, MA, USA). The total time of analysis for each run was 10 min. The column temperature was maintained at 25 °C, the UV detection wavelength was set at 254 nm and the injection volume was 50 μL. Theophylline was used as the internal standard (IS).

The concentrations of sorafenib, sorafenib *N*-oxide were assayed using the modified HPLC method with UV detection [30]. Separation was achieved by gradient elution of the mobile phase, comprising ammonium acetate 0.1 M pH 3.4 (adjusted with glacial acetic acid)—eluent A and acetonitrile—eluent B, at a flow rate of 1.0 mL/min through an reversed phase C8 column (Symmetry^®^ C8, 250 mm × 4.6 mm, 5.0 μm particle size) (Waters Corporation^®^, Milford, MA, USA). The total time of analysis for each run was 22 min. Linear gradient started at 60% eluent A and 40% eluent B to 29% eluent A and 71% eluent B. The column temperature was maintained at 25 °C, the UV detection wavelength was set at 265 nm and the injection volume was 20 μL. Lapatinib was used as IS.

### Pharmacokinetic evaluation

The Phoenix^®^ WinNonlin version 8.1 software (Certara L.P.) was used for the calculation of the pharmacokinetic parameters based on the plasma concentrations paracetamol, paracetamol glucuronide, paracetamol sulphate, of sorafenib, sorafenib *N*-oxide. The maximum plasma concentration (*C*_max_) and the time to reach the *C*_max_ (*t*_max_) were derived directly from the observed plasma concentrations. The total area under the concentration–time curve (AUC) was estimated by the trapezoidal rule with extrapolation from time zero to infinity (AUC_0-∞_). The elimination half-life (*t*_0.5_) was estimated from ln2/*k*_el_. The apparent plasma drug clearance (Cl/F) was calculated by dividing the dose (*D*) by the AUC_0-∞_. The apparent volume of distribution (*V*_d_/*F*) was estimated from *D*/(*k*_el_ × AUC_0-∞_).

### Statistical analysis

The traits were tested for departure from normality using the Shapiro–Wilk test. The traits which did not show significant deviation from normality were subject to the heterogeneity of variance test, followed by pooled (heterogeneity of variance test *p*-value > 0.05) or Satterthwaite (heterogeneity of the variance test *p*-value < 0.05) *t*-tests to verify the significance of differences between the *I*_S+PA_ and II_S_ or *I*_S+PA_ and III_PA_. Differences between the *I*_S+PA_ and II_S_ or I_S+PA_ and III_PA_ in the characteristics which showed significant deviation from normality were tested with the Kruskal–Wallis test. The analysis was performed using capability, t-test and npar1way procedures of SAS (SAS Institute Inc. 2002–2012. The SAS System for Windows version 9.4. Cary, NC, USA).

## Results

The calibration curve for paracetamol was linear within the range of 1.0–60.0 µg/mL (*r* = 0.999), for paracetamol glucuronide within the range of 0.2–50.0 µg/mL (*r* = 0.999), and for paracetamol sulphate within the range of 0.2–60.0 µg/mL (*r* = 0.998). The high precision (coefficient of variation, CV < 10%) and accuracy (%bias ≤ 6.0%) for paracetamol, paracetamol glucuronide and paracetamol sulphate of the applied methodology was obtained.

The calibration curve for sorafenib was linear within the range of 0.025–5.0 µg/mL (*r* = 0.998) and for sorafenib *N*-oxide within the range of 0.02–0.40 µg/mL (*r* = 0.999). The lower limit of quantification (LLOQ) for sorafenib and sorafenib *N*-oxide were 0.025 and 0.020 µg/mL, respectively. The high precision (coefficient of variation, CV < 12%) and accuracy (%bias ≤ 7.5%) of the applied methodology was confirmed for both analytes. The retention times for lapatinib, sorafenib *N*-oxide, sorafenib were 9.0, 12.8, 15.6 min, respectively. The relative recovery for sorafenib, sorafenib *N*-oxide, lapatinib were 92.1, 87.3, 52.0%, respectively.

All the data were expressed as the mean value ± standard deviation (SD). The groups of rats did not differ significantly in terms of body mass. Four samples were not collected from group III_PA_ and two samples were not collected from group *I*_SR+PA_ to assay paracetamol, paracetamol glucuronide and paracetamol sulphate. Two samples were not collected from group II_SR_ and three samples were not collected from group *I*_S+PA_ to assay sorafenib and sorafenib *N*-oxide. At times 0.5, 1 and 96 h the levels of sorafenib *N*-oxide were below the LLOQ. The levels of sorafenib *N*-oxide were below the LLOQ in 6 samples at time 2 h.

### The influence of sorafenib on the pharmacokinetics of paracetamol, paracetamol glucuronide and paracetamol sulphate

**Fig. 1:**
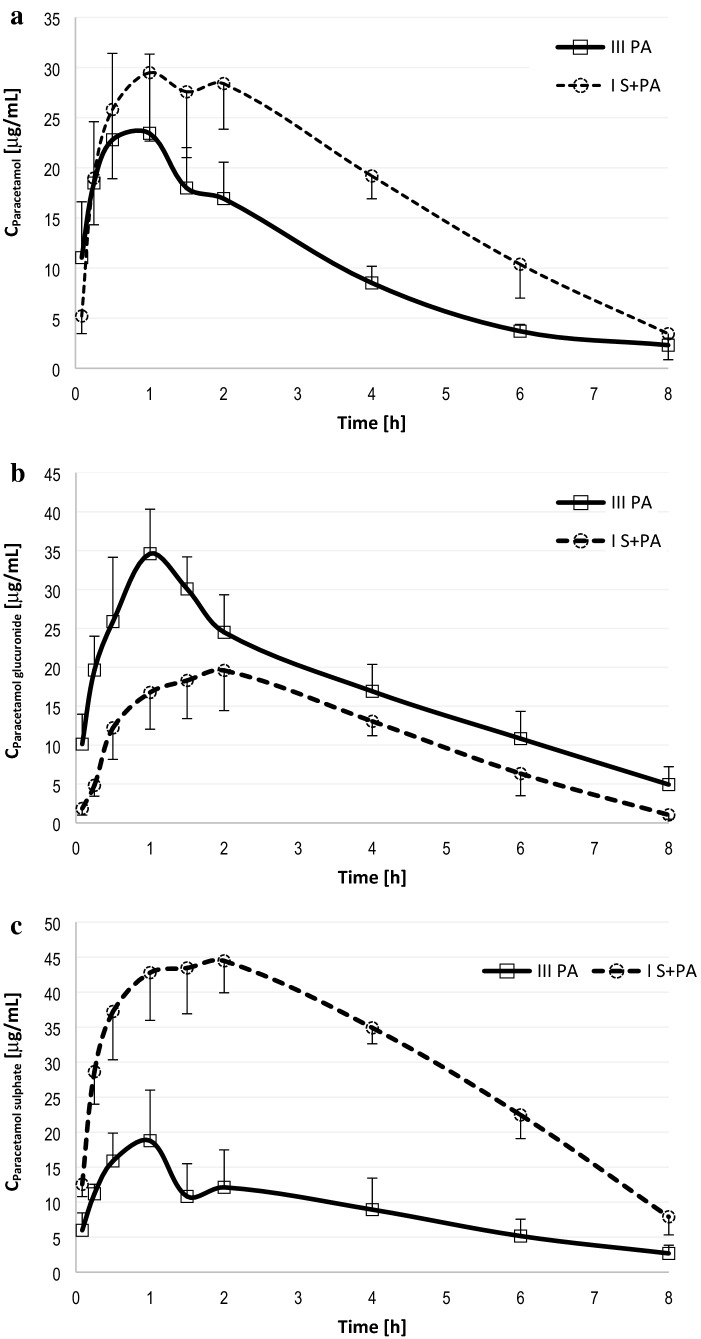
Plasma concentration–time profiles (Mean ± SD) in rats receiving paracetamol (III_PA_) and sorafenib + paracetamol (*I*_S+PA_) of paracetamol (**a**), paracetamol glucuronide (**b**) and paracetamol sulphate (**c**)

The arithmetic means of plasma concentrations for paracetamol and its metabolites: glucuronide and sulphate after oral administration to the groups are shown in Fig. 1. The main pharmacokinetic parameters from non-compartmental methods are summarized in Table 1.

**Table 1:** Plasma pharmacokinetic parameters for paracetamol, paracetamol glucuronide and paracetamol sulphate after oral administration of a single dose of paracetamol (100 mg/kg b.w.) to the III_PA_ group and paracetamol + sorafenib (100 mg/kg b.w. + 100 mg/kg b.w.) to the *I*_S+PA_ group

Pharmacokinetics parameters^a^	III_PA_ (*n* = 8)	*I*_S+PA_ (*n* = 8)	G_mean_ ratio^b^ (90% CI) *I*_S+PA_ vs. III_PA_
Paracetamol			
*C*_max_ (µg/mL)	24.70 ± 8.429 (34)	32.81 ± 5.728 (18)	1.36 (1.07; 1.73)
AUC_0-t_ (µg × h/mL)	80.46 ± 12.10 (15)	140.5 ± 22.13 (16)	1.75 (1.50; 2.03)
AUC_0-∞_ (µg × h/mL)	88.62 ± 8.956 (10)	151.8 ± 30.46 (20)	1.69 (1.46; 1.96)
*t*_max_ (h)	0.8125 ± 0.5786 (71)	1.429 ± 0.6075 (43)	1.99 (1.10; 3.60)
*k*_a_ (h^−1^)	2.351 ± 0.9971 (42)	2.097 ± 1.112 (53)	0.85 (0.50; 1.43)
*t*_0.5_ (h)	2.264 ± 0.7022 (31)	1.661 ± 0.8909 (54)	0.71 (0.51; 0.97)
Cl/F (L/h × kg)	0.5691 ± 0.0560 (10)	0.3406 ± 0.0816 (24)	0.59 (0.50; 0.68)
*V*_d_/*F* (L/kg)	1.981 ± 0.6253 (32)	0.7751 ± 0.2959 (38)	0.39 (0.29; 0.52)
Paracetamol glucuronide			
*C*_max_ (µg/mL)	38.38 ± 3.747 (10)	19.83 ± 5.538 (28)	0.50 (0.41; 0.61)
AUC_0-t_ (µg × h/mL)	136.2 ± 23.58 (17)	87.86 ± 21.75 (25)	0.64 (0.52; 0.77)
AUC_0-∞_ (µg × h/mL)	154.1 ± 35.58 (23)	89.57 ± 22.85 (26)	0.58 (0.46; 0.73)
Paracetamol glucuronide/paracetamol^c^			
*C*_max_	1.667 ± 0.4126 (25)	0.6391 ± 0.2729 (43)	0.37 (0.27; 0.51)
AUC_0-t_	1.748 ± 0.4736 (27)	0.6500 ± 0.2325 (36)	0.36 (0.27; 0.49)
AUC_0-∞_	1.983 ± 0.6384 (32)	0.6278 ± 0.2507 (40)	0.31 (0.22; 0.44)
Paracetamol sulphate			
*C*_max_ (µg/mL)	19.41 ± 7.054 (36)	49.02 ± 4.125 (8)	2.68 (2.05; 3.51)
AUC_0-t_ (µg × h/mL)	71.92 ± 31.04 (43)	242.2 ± 23.63 (10)	3.67 (2.68; 5.02)
AUC_0-∞_ (µg × h/mL)	82.42 ± 32.71 (40)	267.1 ± 34.37 (13)	3.47 (2.58; 4.67)
Paracetamol sulphate/paracetamol^d^			
*C*_max_	0.8764 ± 0.4618 (53)	1.542 ± 0.3434 (22)	1.97 (1.33; 2.91)
AUC_0-t_	0.9142 ± 0.4320 (47)	1.756 ± 0.2738 (16)	2.11 (1.50; 2.96)
AUC_0-∞_	1.046 ± 0.4509 (43)	1.809 ± 0.3431 (19)	1.85 (1.34; 2.56)

^a^*AUC*_*0-t*_ area under the plasma concentration–time curve from zero to the time of last measurable concentration, *AUC*_*0-∞*_ area under the plasma concentration–time curve from zero to infinity, *C*_*max*_ maximum observed plasma concentration, *t*_*max*_ time to first occurrence of *C*_max_, *t*_*0.5*_ half-life in elimination phase, *Cl/F* clearance (Cl), *V*_*d*_*/F* volume of distribution per kilogram, *k*_*a*_ absorption rate constant, arithmetic means ± standard deviations (SD) are shown with CV (%) in brackets

^b^Ratio of geometric means (*G*_mean_) between groups (%) with the upper and lower bounds of a 90% confidence interval (CI) in the brackets

^c^Ratio of paracetamol glucuronide/paracetamol

^d^Ratio of paracetamol sulphate/paracetamol

Sorafenib significantly increased paracetamol *C*_max_ by 33% (*p* = 0.0372). When paracetamol and sorafenib were coadministered, the AUC_0-∞_ of paracetamol grew from 88.62 to 151.80 μg × h/mL (*p* = 0.0018). In the group of rats receiving the both drugs, paracetamol *t*_max_ was longer when compared to the group receiving paracetamol alone, but there was no statistical significance (*p* = 0.0639). Statistically significant differences were revealed for Cl/F (*p* =  < 0.0001), *V*_d_/*F* (*p* = 0.0005), *t*_0,5_ (*p* = 0.0204).

The *C*_max_ of paracetamol glucuronide tended to be higher in the paracetamol group (38.38 vs. 19.83 μg/mL, *p* =  < 0.0001). The exposure to paracetamol glucuronide was significantly lower in the presence of sorafenib, what was reflected by decreased values of AUC_0-t_ (*p* = 0.0012), AUC_0-∞_ (*p* = 0.0012).

Sorafenib elevated paracetamol sulphate *C*_max_ by 152% (*p* = 0.0012). Statistically significant differences were revealed for AUC_0-*t*_ (*p* = 0.0012), AUC_0-∞_ (*p* =  < 0.0001).

### The influence of paracetamol on the pharmacokinetics of sorafenib and sorafenib *N*-oxide

**Fig. 2:**
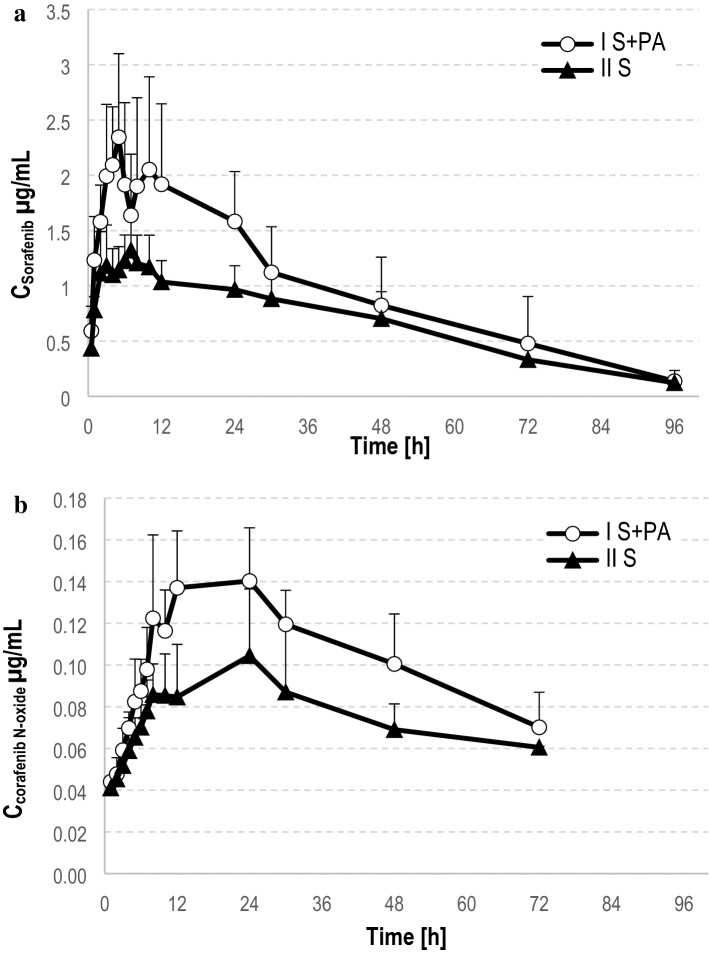
Plasma concentration–time profiles (Mean ± SD) in rats receiving sorafenib (II_S_) and sorafenib + paracetamol (*I*_S+PA_) of sorafenib (**a**), sorafenib N-oxide (**b**)

The arithmetic means of plasma concentrations for sorafenib and sorafenib *N*-oxide after oral administration to the groups are shown in Fig. 2. The main pharmacokinetic parameters from non-compartmental methods are summarized in Table 2.

**Table 2:** Plasma pharmacokinetic parameters of sorafenib and its metabolite N-oxide after oral administration of a single dose of sorafenib (100 mg/kg b.w.) to the II_S_ group and paracetamol + sorafenib (100 mg/kg b.w. + 100 mg/kg b.w.) to the *I*_S+PA_ group

Pharmacokinetic parameters^a^	II_S_ (*n* = 8)	*I*_S+PA_ (n = 8)	*G*_mean_ ratio^b^ (90% CI) *I*_S+PA_ vs. II_S_
Sorafenib			
*C*_max_ (µg/mL)	1.562 ± 0.353 (23)	2.504 ± 0.7615 (30)	1.58 (1.25; 1.99)
AUC_0-t_ (µg × h/mL)	62.83 ± 16.14 (26)	91.44 ± 42.47 (46)	1.39 (1.02; 1.88)
AUC_0-∞_ (µg × h/mL)	67.02 ± 16.70 (25)	95.93 ± 46.15 (48)	1.36 (0.99; 1.86)
*t*_max_ (h)	5.125 ± 2.167 (42)	5.625 ± 1.923 (34)	1.16 (0.81; 1.64)
* k*_a_ (h^−1^)	0.7392 ± 0.3141 (43)	0.7399 ± 0.2336 (32)	0.92 (0.62; 1.35)
*t*_0.5_ (h)	21.89 ± 7.787 (36)	20.80 ± 3.150 (15)	0.96 (0.80; 1.16)
Cl/F (L/h × kg)	0.7986 ± 0.2172 (27)	0.5987 ± 0.2317 (39)	0.72 (0.52; 0.98)
*V*_d_/*F* (L/kg)	25.30 ± 11.59 (46)	17.47 ± 6.271 (36)	0.69 (0.50; 0.94)
Sorafenib *N*-oxide			
*C*_max_ (µg/mL)	0.1133 ± 0.0247 (22)	0.1656 ± 0.0281 (17)	1.47 (1.25; 1.74)
AUC_0-t_ (µg × h/mL)	4.102 ± 1.562 (38)	7.487 ± 1.170 (16)	1.95 (1.45; 2.61)
AUC_0-∞_ (µg × h/mL)	8.609 ± 2.189 (25)	12.42 ± 3.783 (31)	1.89 (1.32; 2.71)
*t*_max_ (h)	16.38 ± 8.210 (50)	15.50 ± 7.231 (47)	0.98 (0.62; 1.54)
*t*_0.5_ (h)	53.31 ± 25.23 (47)	45.57 ± 16.44 (36)	1.59 (1.02; 2.49)
Sorafenib *N*-oxide/sorafenib^c^			
*C*_max_	0.0748 ± 0.0200 (27)	0.0709 ± 0.0241 (34)	0.96 (0.76; 1.20)
AUC_0-t_	0.0669 ± 0.0249 (37)	0.0960 ± 0.0425 (44)	1.40 (0.95; 2.07)
AUC_0-∞_	0.1361 ± 0.0518 (38)	0.1536 ± 0.0758 (49)	1.39 (0.87; 2.22)

^a^*AUC*_*0-t*_ area under the plasma concentration–time curve from zero to the time of last measurable concentration, *AUC*_*0-∞*_ area under the plasma concentration–time curve from zero to infinity, *C*_*max*_ maximum observed plasma concentration, *t*_*max*_ time to first occurrence of *C*_max_, *t*_0.5_ half-life in elimination phase, *Cl/F* clearance (Cl), *V*_*d*_*/F* volume of distribution per kilogram, *k*_*a*_ absorption rate constant, arithmetic means ± standard deviations (SD) are shown with CV (%) in brackets

^b^Ratio of geometric means (*G*_mean_) between groups (%) with the upper and lower bounds of a 90% confidence interval (CI) in the brackets

^c^Ratio of sorafenib *N*-oxide/sorafenib

Paracetamol significantly increased sorafenib *C*_max_ by 60% (*p* = 0.0068). When paracetamol and sorafenib were coadministered, the AUC_0-∞_ of sorafenib increased from 67.02 to 95.93 μg × h/mL, but the elevation was not statistically significant (*p* = 0.0929). There were no significant differences between groups with respect to the following parameters: AUC_0-t_ (*p* = 0.0742), *t*_0.5_ (*p* = 0.5935), *t*_max_ (*p* = 0.6330), Cl/F (*p* = 0.0986), and *V*_d_/*F* (*p* = 0.0783).

The *C*_max_ of sorafenib *N*-oxide was increased by 83% (*p* = 0.0023) in the *I*_S+PA_ group. Statistically significant differences were revealed for AUC_0-t_ (*p* = 0.0002) and AUC_0-∞_ (*p* = 0.0065). The mean *t*_max_ of sorafenib *N*-oxide was similar in the both groups (16.3 vs. 15.5 h, *p* = 0.9121).

## Discussion

Cancer therapy frequently requires polypharmacy which increases the risk of drug–drug interactions. Patients take over-the-counter drugs as well as alternative medicaments, herbs and dietary supplements, which may also interact with each other. What is more, patients do not always inform physicians about the other drugs they are taking to support regular therapy and improve their overall health condition. According to some studies, on average cancer patients receive 5–8 drugs [31].

### The influence of sorafenib on the pharmacokinetics of paracetamol, paracetamol glucuronide and paracetamol sulphate

As it is necessary to use painkillers in oncological therapy, the likelihood of simultaneous use of sorafenib and paracetamol increases. In view of the fact that studies have proved strong inhibition of some UGTs by sorafenib (UGT1A9 and UGT1A1) [21] and the fact that glucuronidation is one of the metabolic pathways of paracetamol, there is a risk of interaction between these two drugs.

Conjugation with glucuronic acid is the most common mechanism of metabolism of xenobiotics and endogenous compounds (e.g. bilirubin and steroid hormones). This process is catalysed by UDP-glucuronyl transferase enzymes, mostly the UGT1A, UGT2A and UGT2B subfamilies [19]. UGT1A6, UGT1A9, and UGT2B15 participate in the metabolism of paracetamol [32]. Liu et al. [26] investigated the influence of various kinase inhibitors on the paracetamol glucuronidation process in vitro, using recombinant UDP-glucuronyl transferases and liver microsomes. The researchers observed that sorafenib, dasatinib and imatinib inhibited UGT1A9 and UGT2B15—the isoenzymes responsible for paracetamol glucuronidation. Additionally, the FDA and EMA recommend a detailed description of the inhibition of the UGT enzyme by TKIs [21]. The inhibition of drug metabolism by the UGT enzyme causes a wide range of clinically relevant DDIs. Regorafenib and sorafenib are the strongest human inhibitors of UGT enzymes that have been identified so far [21, 33]. Miners et al. [21] conclude that the extrapolation of in vitro*-*in vivo studies indicates that the inhibition of UGT1A1 significantly contributes to hyperbilirubinemia observed in patients treated with sorafenib.

When paracetamol and sorafenib were co-administered, the *C*_max_ and AUC_0-∞_ of paracetamol increased in *I*_S+PA_ group by 33% and 71%, respectively (Table 1). After a single dose of 100 mg/kg of paracetamol administered orally Pingili et al. [34]. observed a lower *C*_max_ (5.04 µg/ml), longer *t*_max_ (1.16 h) and longer *t*_0.5_ (4.43 h). When Mekjaruskul et al. [35] administered an analogous dose orally, they noted a higher *C*_max_ (19.10 µg/ml), longer *t*_max_ (1.0 h) and shorter *t*_0.5_ (21.29 min).

Moreover, the study has revealed the significantly lower exposure to paracetamol glucuronide (decreased *C*_max_ and AUC_0-∞_ by 48% and 42%, respectively) in the presence of sorafenib (Table 1). However, the ratios of paracetamol glucuronide/paracetamol did not reach statistical significance, suggesting the lack of influence of sorafenib on the glucuronidation of paracetamol. Nonetheless, it cannot be ruled out that the lack of significant differences in the paracetamol glucuronide/paracetamol ratios may result from the compensation of the glucuronidation pathway by the UGT1A6 form of the isoenzyme or insufficient power of the experiment. In the *I*_S+PA_ group we also observed increased *C*_max_ and AUC_0-∞_ of paracetamol sulphate by 2.1- and 2.7-fold, respectively with a simultaneous increase in paracetamol sulphate/paracetamol ratio for *C*_max_ and AUC_0-∞_ (*p* = 0.0003 and *p* = 0.0003, respectively). Karbownik et al. [36–38] conducted an in vivo study and proved that other TKIs, i.e. erlotinib, lapatinib and sunitinib significantly affected the PK of paracetamol. After the administration of erlotinib, lapatinib and sunitinib the *C*_max_ of paracetamol dropped by: 18.9%, 55.7% and 63.95% and the AUC_0-∞_ decreased by 35.5%, 48.8% and 68.19%, respectively. That research also proved that all these TKIs inhibited the paracetamol glucuronidation process.

The current analysis of the PK of paracetamol and its metabolites was significantly limited by the lack of NAPQI measurements.

### The influence of paracetamol on the pharmacokinetics of sorafenib and sorafenib *N*-oxide

The concomitant application of sorafenib and paracetamol contributed to 1.6-fold increase of sorafenib *C*_max_. The comparison of the PK data of sorafenib in our project with the data published by Wang [27] and Wang [39] showed that in our study there were higher *C*_max_ values: 1.562 µg/mL, 230.86 µg/L, 338 ng/mL, respectively. The changes in the concentration may have been caused by the drug carrier but they also confirmed the high PK variability. The *T*_max_ in our study was also shorter than in the studies by Wang and Wang (5.125, 8.14, 8.0 h, respectively). We did not compare the AUC data because the blood samples were collected at different times. In our study it was up to 96 h, whereas it was up to 48 h in the study by Wang and up to 36 h in the study by Wang. In our project the *t*_0.5_ was also longer than in the studies by Wang and Wang. Moreover, coadministration of paracetamol increased C_max_ and AUC_0-∞_ of sorafenib active metabolite—*N*-oxide by 55 and 79%, respectively. However, the values of *N*-oxide sorafenib/sorafenib ratios for *C*_max_ and AUC_0-∞_ in the II_S_ and *I*_S+PA_ groups (*p* = 0.5632 and *p* = 0.5952, respectively) did not confirm significant influence of paracetamol on sorafenib oxidation. The increased exposure of sorafenib and its active metabolite is most likely to have been caused by the inhibition of P-gp transport of this drug by paracetamol. Nevertheless, higher concentrations of sorafenib and sorafenib *N*-oxide may improve the response to therapy, but on the other hand potentiate adverse drug reactions. Both of these possibilities should be taken into account during therapy. These findings deserve further study and confirmation in humans.

As the resulting direction and extent of this interaction may be individual and influenced by dosing schedule, the randomized clinical trials after single and multiple doses of paracetamol are required.

The lack of differences in the values of the *k*_a_ constant parameters of paracetamol and sorafenib (2.35 ± 0.99 vs. 2.09 ± 1.11 h^−1^ (*p* = 0.6089); 0.81 ± 0.32 vs. 0.74 ± 0.23 h^−1^ (*p* = 0.6110), respectively) shows that the drugs did not influence the absorption rate of each other.

Karbownik et al. examined the influence of paracetamol on the pharmacokinetics of lapatinib and erlotinib [36, 37]. The co-administration of lapatinib and paracetamol increased the AUC_0-t_ and *C*_max_ of lapatinib by 240% (*p* = 0.0030) and by 184% (*p* = 0.0011), respectively. When erlotinib was applied concomitantly with paracetamol, the AUC_0-t_ and *C*_max_ also increased (by 31%, *p* = 0.0329 and by 88%, *p* = 0.0004, respectively). The research results indicated that the drugs used in oncological therapy may increase the intensity of adverse reactions, including those reducing the quality of life, e.g. diarrhoea.

The selection of analgesic drug is an important aspect during the sorafenib therapy of HCC patients. Li et al. [40] proved that the combination therapy with acetylsalicylic acid (ASA) and sorafenib gave a synergistic anticancer effect against liver tumours both in vitro and in vivo. The combination of these drugs induced apoptosis in the tumours without causing a weight loss, hepatotoxicity or inflammation. The authors have suggested that ASA overcomes the resistance to sorafenib and the combination of the drugs may be an effective approach to improve HCC treatment. Fujioka et al. [41] observed that the administration of a strong opioid (morphine) for anesthesia may promote the progression of lung cancer in the EGFR (epidermal growth factor receptor) phosphorylation pathway. This phenomenon decreases the efficacy of drugs, e.g. erlotinib. Therefore, the choice of the analgesic drugs in cancer patients should be based not only on its potency to reduce pain, but also on the possible influence on the activity and efficacy of anticancer drug.

The study was significantly limited by the lack of assay of sorafenib glucuronide and the free fraction of sorafenib. The free fraction of the drug was not measured because sorafenib binds to human plasma proteins in 99.8% in an in vitro study [42].

## Conclusion

After single doses of paracetamol and sorafenib we observed increased exposure to paracetamol. Although we have not confirmed the inhibition of paracetamol glucuronidation by sorafenib, the study revealed the inducing effect of sorafenib on paracetamol sulfation. Moreover, the coadministration of sorafenib and paracetamol may result in increased sorafenib concentrations and improved exposure to its active N-oxide metabolite. These changes may contribute to better response to sorafenib therapy or intensification of adverse effects of the drug. A greater knowledge of DDIs between sorafenib and paracetamol may help adjust dose properly and avoid toxicity effects in individual patients. Nonetheless, all the obtained results should be confirmed by clinical trials on patients, including clinical assessment of response to treatment.
